# Sodium dodecylsulfate-layered double hydroxide and its use in the adsorption of 17β-estradiol in wastewater

**DOI:** 10.1039/c8ra05726e

**Published:** 2018-09-06

**Authors:** Yuan Kong, Yangrui Huang, Chenrui Meng, Zhi Zhang

**Affiliations:** Key Laboratory of the Three Gorges Reservoir Region's Eco-Environment, Ministry of Education, Chongqing University Chongqing 400045 China zhangzhicqu@cqu.edu.cn; School of Environmental Studies, China University of Geosciences Wuhan 430074 Hubei China

## Abstract

Modified Mg_3_Al layered double hydroxide (LDH) intercalated with dodecylsulfate anion composites, which were designated as SDS-LDH composites, were synthesized by coprecipitation. The samples were characterized using SEM, EDX, FT-IR, zeta potential analysis, and XRD. The results showed that the SDS-LDH composites contain a thicker and larger porous interconnected network than inorganic LDH due to the enlarged inter-layer distance. The outstanding adsorption performance of SDS-LDH composites toward 17β-estradiol (E2) was investigated under different conditions, including solution pH, adsorbent dosage, ion strength, reaction time, and temperature. When the solution pH was 7 and the adsorbent dosage was 2 g L^−1^, the removal rate of E2 reached the maximum at 94%, whereas inorganic LDH displayed a poor E2 removal rate of 10%. The presence of various ions (Na^+^, SO_4_^2−^, CI^−^, and H_2_PO_4_^−^) in aqueous solution exerted no significant adverse effects on the adsorption process. The adsorption equilibrium was reached within 20 min, and the adsorption fitted well with the pseudo-second-order model and the Freundlich isotherm. The thermodynamic test revealed that the adsorption process was spontaneous and endothermic. Phosphorus was selected as the index for evaluating the adsorption capacity of SDS-LDH composites for inorganic ions. The removal rates of total phosphorus and PO_4_^3−^ were 43.71% and 55.93% for SDS-LDH composites at 2 g L^−1^. The removal rate of PO_4_^3−^ reached up to 85% when the contact time was 120 min and the dosage was 3 g L^−1^ for SDS-LDH composites, which were approximately close to those of inorganic LDH of 30 min and 2 g L^−1^, respectively. This finding indicates that the removal capacity of SDS-LDH composites for PO_4_^3−^ decreased after the dodecylsulfate anions intercalated into the interlayer. The composites retained their high efficiency and stability after desorption and regeneration with alkali treatment. This study demonstrated that SDS-LDH composites are a promising adsorbent for the recovery and abatement of trace-level E2 in secondary effluents of wastewater treatment plants.

## Abstract 2

SDS-LDH composites were synthesized by coprecipitation. The composites are promising adsorbents for the recovery and abatement of trace-level E2 in secondary effluents of wastewater treatment plants.

## Graphical abstract

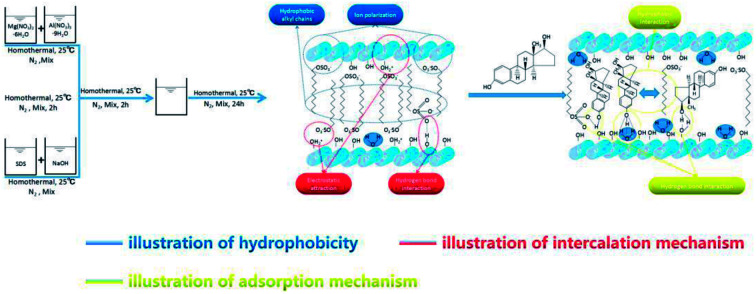

## 1 Introduction

The abundance of environmental estrogens, particularly endocrine-disrupting compounds, which are considered emerging persistent compounds, is potentially endangering the ecological health in environmental water and affecting the normal activity of the endocrine system in both wildlife and humans.^1^ Among these compounds, the natural hormone 17β-estradiol (E2) is a highly endocrine-disrupting agent. On the one hand, E2 is unlikely the most probable cause of endocrine disruption effects in fish and other animal species.^2^ On the other hand, E2 is associated with endocrine and reproductive complications, including uterine fibroids, breast cancer in women, and testicular and prostate cancers in men.^1,3,4^ E2 has been detected in different types of water, including drinking water, natural water bodies, and wastewater treatment plant effluents and sludge throughout Europe, Asia, and the United States.^5,21^ The E2 levels in groundwater reportedly range from below 1 ng L^−1^ to 64 ng L^−1^, and E2 exhibits a high physiological activity even at very low concentrations. The removal rate of environmental estrogens, including E2, by municipal wastewater treatment plants ranges from 50% to 90%.^6^ In environmental water, the degradation of E2 and related compounds typically lasts for numerous hours to months or even several years. Various methods, such as photocatalytic degradation, zonation, activated sludge, and adsorption, have been established to remove environmental estrogens. For example, Silva C. P. *et al.*^7^ used simulated solar radiation to investigate the photodegradation of 17α-ethinylestradiol (EE2) and E2 in the presence of fulvic acids. Coleman *et al.*^8^ investigated the removal efficiency of E2 by adding powdered TiO_2_ into a photocatalytic degradation system. Irmak *et al.*^9^ explored the treatment efficiency of E2 in ozone (O_3_) and O_3_/ultraviolet (UV) systems. However, the treatment efficiency is restricted by external conditions, and the cost is always very high. In contrast to these methods, adsorption has become the most promising technique because of its easy synthesis, low cost, and high efficiency. For example, Fukuhara T. *et al.*^10^ investigated the adsorption performance of estrone and estradiol dissolved in ultra-pure water by using activated carbon, and they obtained remarkable results. Yoon Y. *et al.*^11^ investigated the adsorption performance of ^3^H-labeled E2 by using powdered activated carbon and obtained maximum final removal rates up to 90%. Unfortunately, when traditional adsorbents are applied, competitive adsorption may adversely affect the adsorption process.

Layered double hydroxides (LDHs) consist of positively charged brucite-type layers of divalent and trivalent metallic hydroxide and exchangeable anions located in the interlamellar that are used to balance the positive charge, and LDHs are considered a typical anionic clay.^11–13^ The unusual physicochemical properties of LDHs are due to the unique lamellar structure and wide combination range of the components of both layers and interlayers. Given their specific properties, such as surface adsorption, surface complexation, ion exchange, and memory effect, LDH composites are regarded as promising materials for adsorbing pollutant.^14^ Traditional LDHs synthesized by coprecipitation or ion exchange are hydrophilic, resulting in a low affinity for non-ionic organic compounds.^1^ However, the hydrophobic interaction between LDH composites and hydrophobic pollutants could be improved by replacing the interlamellar components with specific anions to enhance the affinity toward hydrophobic pollutants.^15,16^ These modified LDHs have a wide range of applications in the control of organic pollution fields.^17–19^

In these experiments, we used coprecipitation to modify layered double hydroxides intercalated with dodecylsulfate anions, which were designated as SDS-LDH composites.^19^ The formed materials were systematically characterized by scanning electron microscopy (SEM), energy dispersive X-ray (EDX), Fourier transform infrared spectroscopy (FT-IR), Zeta potential and X-ray diffraction (XRD) to evaluate their adsorption efficiency and clarify the adsorption mechanism of E2. Then, adsorption experiments were conducted under different conditions, including solution pH, adsorbent dosage, ion strength, contact time, and temperature. The adsorption kinetics, adsorption isotherm, and adsorption thermodynamics of E2 were analyzed to elucidate the adsorption mechanism. Cyclic adsorption experiments were performed to evaluate the practicability of the adsorbents and improve the process economics. Phosphorus were used to evaluate the change in adsorption capacity for inorganic matter after modification. Corresponding experiments involved the removal rates of TP (total phosphorus) and PO_4_^3−^ by SDS-LDH composites with 2 g L^−1^ and the comparison of removal efficiency of PO_4_^3−^ between SDS-LDH composites and inorganic LDH. In view of the effects of E2 on environmental systems, our findings are critical to understanding the key factors influencing natural photodegradation processes. The results of this study may aid in the management of this environmental stressor.

## 2 Materials and methods

### 2.1 Materials

The 17α-estradiol and E2 used in this study were purchased from J&K in powder form with a purity of 99%. Their molecular structure and relevant physicochemical properties are summarized in Table 1.^20^ The organic anion dodecylsulfate used in preparing the organo/LDH nanohybrid was purchased from Sinopharm Chemical Reagent Co., Ltd. with a >99% purity. All other solvents and reagents (Sinopharm Chemical Reagent Co., Ltd.) were of analytical grade and used without further purification. The wastewater used in all experiments was the filtered secondary effluent of the Long Wang Zui Wastewater Treatment Plant.

**Table 1:** Molecular structure and physicochemical properties of 17α-estradiol and E2

Molecule	Chemical formula	Molecule structure	MW (g moL^−1^)	Water solubility (mg L^−1^)	l g *K*_ow_	p*K*_a_
17α-Estradiol	C_18_H_24_O_2_	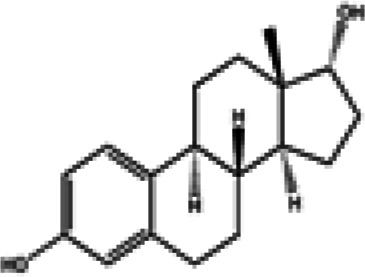	272.38	13	3.94	10.46
17β-Estradiol	C_18_H_24_O_2_	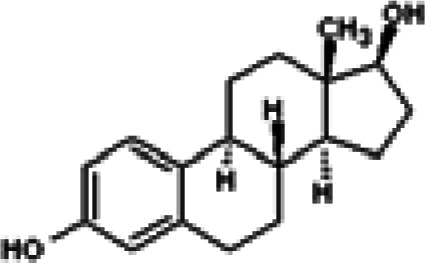	272.38	13	3.94	10.71

### 2.2 Preparation of SDS-LDH and inorganic LDH composites

**Scheme 1:**
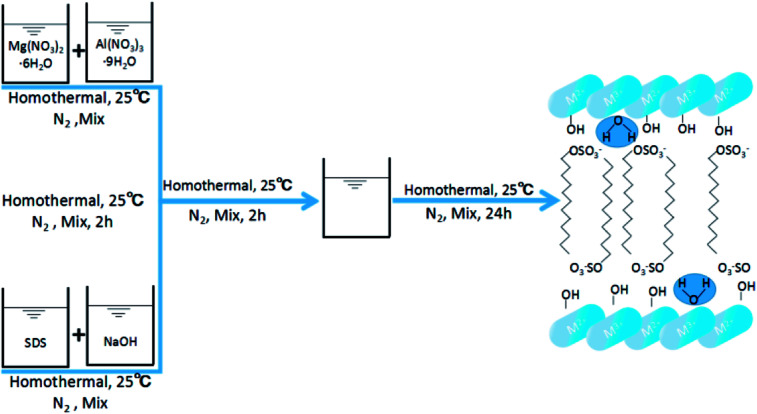
Synthetic routes and schematic illustrations of SDS-LDH composites.

SDS-LDH composites were synthesized by coprecipitation under N_2_ atmosphere and CO_2_-free water.^12^ A 100 mL aqueous solution labeled as “solution A” contained 0.06 mol of Mg(NO_3_)_2_·6H_2_O and 0.02 mol of Al(NO_3_)_3_·9H_2_O (Mg/Al ratio = 3), and another 500 mL alkaline solution denoted as “solution B” contained 0.16 mol of NaOH and 0.05 mol of sodium dodecylsulfate. Solution A was added dropwise to solution B. Then, the mixture was homothermally treated at 80 °C for 24 h and aged. The suspension was centrifuged at a high speed and washed with CO_2_-free water for several times. Finally, the samples were freeze dried, and the SDS-LDH composites were obtained. The synthetic routes and schematic illustrations of the SDS-LDH composites are presented in Scheme 1.

For comparison, inorganic LDH was prepared by coprecipitation.^12^ A 100 mL aqueous solution labeled as “solution C” contained 0.075 mol of Mg(NO_3_)_2_·6H_2_O and 0.025 mol of Al(NO_3_)_3_·9H_2_O (Mg/Al ratio = 3), and another 500 mL alkaline solution designated as “solution D” contained 0.2 mol of NaOH and 0.0375 mol of Na_2_CO_3_. Solution C was added dropwise to solution D. Then, inorganic LDH was obtained through the same process as described.

### 2.3 Characterization

The SDS-LDH composites and inorganic LDH composites were characterized using different physicochemical techniques. SEM images were captured using a SU8010 (Hitachi Co., Japan) instrument at a voltage of 1 kV and a resolution of up to 1.3 nm. Elemental chemical analysis was conducted through atomic absorption spectrometry on an APOLLO XP (Ametek Co., USA) instrument. EDX spectroscopy revealed the main elementals analysis results, including the weight (%) and atomic ratio of the SDS-LDH and inorganic LDH composites. FT-IR was performed using a Nicolet6700 (Bruker, USA) instrument. The XRD diagrams were collected at room temperature under air conditions by using a Bruker AXS D8-Focus X-ray diffractometer instrument with Cu K_α_ radiation (*λ* = 0.154050 nm) and quartz as the external standard. The interlamellar pacing was calculated using the following equation:1
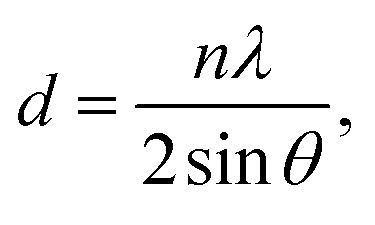
where *d* (nm) is the interlamellar spacing, *n* (*n* = 1) is the diffraction series, *λ* (*λ* = 0.154050 nm) is the wavelength of the X-ray, and *θ* represents the half of the diffraction angle.

### 2.4 Analysis methods

E2 was analyzed by high-performance liquid chromatography (HPLC). The chemicals were separated on a Waters PAH C-18 (S-5 μm, 250 mm × 4.6 mm column) with 60 : 40 acetonitrile/water as the mobile phase. The equipment was run for 10 min at a flow rate of 0.8 mL min^−1^. A 20 μL injection volume of E2 was analyzed by HPLC with the use of a UV detector at 279 nm and a fluorescence detector operating simultaneously in series. The concentrations of PO_4_^3−^ and TP in the samples were measured by the ascorbic acid–molybdenum blue method and the potassium persulfate digestion–ascorbic acid–molybdenum blue method, respectively. For all extractions, after treatment, the sample was centrifuged and passed through a 0.45 μm filter to separate the extract from the residue.^12^

The adsorption amounts *q*_t_ (mg g^−1^) were calculated using the following equation:2
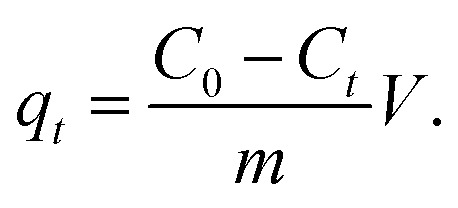


The removal rates were calculated using the following equation:3
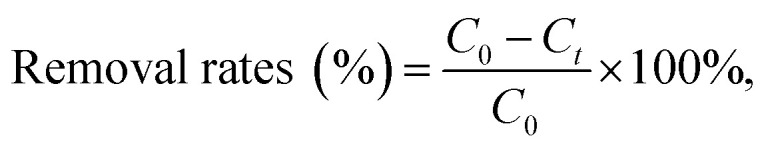
where *C*_0_ (mg L^−1^) is the initial concentration of E2, TP, PO_4_^3−^, and organophosphorus; *C*_*t*_ (mg L^−1^) is the concentration of E2, TP, PO_4_^3−^, and organophosphorus at time *t* (min); *V* (L) is volume of the solution; and *m* (mg) is the weight of the adsorbent.

### 2.5 Adsorption experiments

#### 2.5.1 Preparation of the main solutions

The mother solution of 17α-estradiol (1000 ppm), which was marked as “MA”, was prepared by dissolving 1.00 g of white powdery 17α-estradiol in 1000 mL of methanol and was used as an internal standard. The mother solution of E2 (1000 ppm), which was marked as “MB”, was prepared by dissolving 1.00 g of white powdery 17β-estradiol in 1000 mL of methanol. The stock solutions were covered with aluminum foil to prevent any photodegradation at 4 °C. The mother solution of KH_2_PO_4_ (1000 ppm) was labeled as “MP” and the concentration of MP was calculated by the amount of P being 1000 ppm, so “MP” was prepared by weighing 4.394 g of KH_2_PO_4_, which was dissolved in ultra-pure water and transferred to a volumetric flask (1000 mL).

#### 2.5.2 Adsorption reaction of E2 and phosphorus

The adsorption experiments toward E2 was investigated under different conditions, including solution pH, adsorbent dosage, ion strength, contact time, and temperature. The aqueous solution used in this experiment was the secondary effluent of a wastewater treatment plant with a certain amount of MB. Prior to the adsorption experiments, both the SDS-LDH composites and inorganic LDH composites were dried in a freeze dryer. To reach the adsorption equilibrium, the mixtures was stirred with a magnetic stirrer at 200 rpm for 45 min.

The effect of pH was investigated by adjusting the initial pH of the solutions with 0.1 mol L^−1^ HCl and 0.1 mol L^−1^ NaOH, and the pH range was from 3 to 10. Approximately 0.06 mL of MB was added to 200 mL of filtered sewage. The solution volume, dosage, *C*_0_, and temperature were 200 mL, 2 g L^−1^, 0.319 mg L^−1^, and 298 K, respectively. The effect of adsorbent dosage was assessed by adding 0.06 mL of MB to 200 mL of filtered sewage with different SDS-LDH composites dosages ranging from 0.2 g L^−1^ to 3.5 g L^−1^. The solution volume, pH, *C*_0_, equilibrium time, and temperature were 200 mL, 7, 0.319 mg L^−1^, 45 min, and 298 K, respectively. Then, the adsorption isotherm experiments were repeated at 308 and 318 K. The effect of ion strength was examined by gradually increasing the Na_2_SO_4_, NaCl, and NaH_2_PO_4_ concentrations from 2 g L^−1^ to 10 g L^−1^. Approximately 0.06 mL of MB was added to 200 mL of filtered sewage. The solution volume, pH, dosage, *C*_0_, equilibrium time, and temperature were 200 mL, 7, 2 g L^−1^, 0.319 mg L^−1^, 45 min, and 298 K, respectively. The effect of temperature was analyzed at 298 K by adding 2 g L^−1^ of adsorbents into aqueous solution (200 mL, pH = 7) at different initial concentrations (0.301, 0.275, and 0.260 mg L^−1^) for 45 min. Then, the adsorption isotherm experiments were repeated at 308 and 318 K.

The adsorption experiments toward phosphorus was to evaluating the adsorption capacity of SDS-LDH composites for inorganic ions. Corresponding experiments involved the removal rates of TP and PO_4_^3−^ by SDS-LDH composites with 2 g L^−1^ and the comparison of removal efficiency of PO_4_^3−^ between SDS-LDH composites and inorganic LDH. The aqueous solution of these experiment was the filtered secondary effluent of a wastewater treatment plant added with a certain amount of MP. Prior to the adsorption experiments, both the SDS-LDH composites and inorganic LDH composites were dried in a freeze dryer. Based on the pre-experiment results, the mixtures were stirred with a magnetic stirrer at 200 rpm for 3 h to reach the adsorption equilibrium.

To examine the removal rates of TP, PO_4_^3−^ by SDS-LDH composites, we added 0.40 mL of MP to 200 mL of filtered sewage. The solution volume, pH, dosage, equilibrium time, temperature, and C_0_ of TP and PO_4_^3−^ were 200 mL, 7, 2.0 g L^−1^, 3 h, 298 K, 2.952, and 2.379 mg L^−1^, respectively. The samples were double extracted at different times in which one sample was directly measured without digestion. The measurement of dissolved phosphorus was consistent with the aforementioned method. To evaluating the adsorption capacity of SDS-LDH composites for inorganic ions, we compared the adsorption efficiency of PO_4_^3−^ by SDS-LDH composites (2.0–3.0 g L^−1^) and inorganic LDH (2.0 g L^−1^), and the solution volume, pH, *C*_0_, and temperature were 200 mL, 7, 2.378 mg L^−1^, and 298 K, respectively.

### 2.6 Adsorption kinetics

To study the adsorption kinetics of SDS-LDH composites and inorganic LDH composites for E2, 0.15 mL of MB was added to 500 mL filtered sewage and performed the experiments under different conditions. The solution volume, pH, dosage, *C*_0_, equilibrium time, and temperature were 200 mL, 7, 2 g L^−1^, 0.319 mg L^−1^, 45 min, and 298 K, respectively. The samples were extracted at different times and analyzed.

The nonlinear forms of the pseudo-first-order and pseudo-second-order kinetic models are expressed as the following equations:

4 *q*_*t*_ = *q*_e_(1 − e^−*k*_1_*t*^),

5
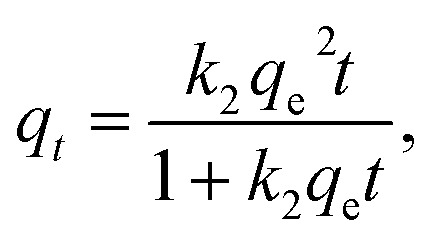
where *q*_e_ (mg g^−1^) is the equilibrium adsorption amounts; *q*_*t*_ (mg g^−1^) is the adsorption amount varying with *t*; *t* (s) is the contact time; and *k*_1_ (s^−1^) and *k*_2_ (g mg^−1^ s^−1^) are the rate constants of the pseudo-first-order and pseudo-second-order models, respectively.

The linear forms of the Langmuir and Freundlich models are given as follows:6
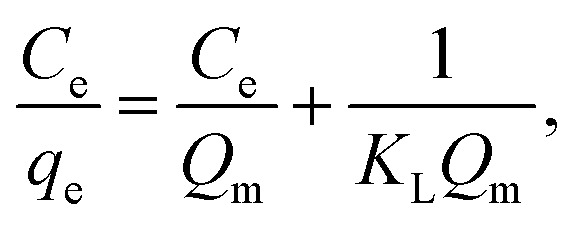
7
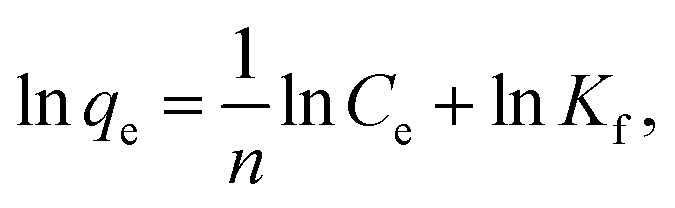
where *q*_e_ (mg g^−1^) is the equilibrium adsorption amount; *C*_e_ (mg L^−1^) is the equilibrium adsorption concentration; *Q*_m_ (mg g^−1^) is the maximum adsorption amount corresponding to the complete monolayer converge; *K*_L_ (L mg^−1^) is the Langmuir constant related to the adsorption energy and the adsorption capability; and *K*_f_ (mg^1−1/*n*^ L^1/*n*^ g^−1^) and *n* are the Freundlich constants representing the adsorption capacity and the adsorption intensity, respectively. Fig. 12 and 13 show the adsorption isotherm curves of E2 under different temperatures and the corresponding linear fitting of the Langmuir and Freundlich (inset) models.

The apparent equilibrium constant (*K*_0_) can be expressed as follows:8
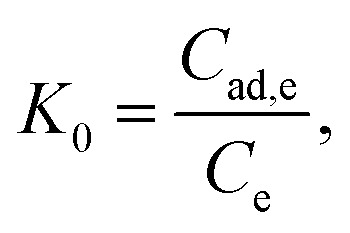
where *C*_ad,e_ (mg) is the equilibrium amount of E2 adsorbed on the adsorbent per liter of the solution, and *C*_e_ (mg L^−1^) is the equilibrium concentration. *K*_0_ can be obtained in the lowest experimental concentration of E2 and used to determine Δ*G*, Δ*H*, and Δ*S* by using the following equations:

9 Δ*G* = −*RT* ln *K*_0_,

10 Δ*G* = Δ*H* − *T*Δ*S*,

where Δ*G* (kJ mol^−1^) is the Gibbs free energy change, Δ*H* (kJ mol^−1^) is the enthalpy change, and Δ*S* (J mol^−1^ K^−1^) is the entropy change.

### 2.7 Cyclic adsorption experiments

The desorption principle was adopted to explore the regeneration performance of SDS-LDH. The cycle adsorption experiments were divided into four groups. The NaOH concentrations used to wash the materials in each group were 2%, 5%, 8%, and 10%, and the adsorption–regeneration cycles were repeated five times for each NaOH concentration. For each adsorption test, the adsorbent dosage was 2 g L^−1^, the external E2 dosage was 0.3 ppm, and the initial pH was 7. After being stirred in a 25 °C water bath and reacted for 30 min, the water sample was collected for further testing.

## 3 Results and discussion

### 3.1 Characterization of the SDS-LDH and inorganic LDH composites

**Fig. 1:**
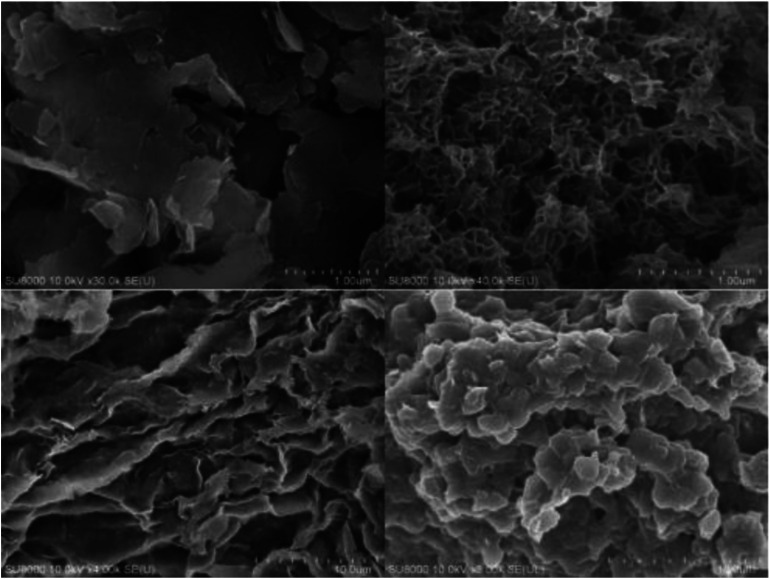
SEM of inorganic LDH composite (left) and SDS-LDH composites (right).

The means of the SEM measurements were used to observe the microstructural surface of the materials, and the results are shown in Fig. 1. As shown, both SDS-LDH composites (right) and inorganic LDH composites (left) consisted of nanosheets, which is typical for layered double hydroxides. In contrast to the inorganic LDH composites, SDS-LDH composites contained a porous interconnected network that is thicker than that of inorganic LDH composites due to the enlarged inter-layer distance. The SDS-LDH composites displayed a typical spreading lamellar structure, and its layers were thin and diffused irregularly.

Table 2 provides the main elementals analysis results, including weight (%) and the atomic ratios of SDS-LDH and inorganic LDH. As shown in Table 2, elemental analysis revealed no detectable amounts of N after dodecylsulfate was intercalated into the interlayer, indicating the lack of NO_3_^−^ in the interlayer. Both the weight (%) and atomic ratio of C and S were markedly increased, suggesting that dodecylsulfate replaced CO_3_^2−^ and NO_3_^−^ into the interlayer. The molar ratio of S/AI was 1.40, which was higher than the required amount (S/AI = 1) to replace the inorganic anions in the interlayer. Other researchers have also reported this phenomenon. You *et al.*^22^ found that the excessive dodecylsulfate intercalated into the interlayer could be due to non-polar interactions with the hydrophobic alkyl groups of the intercalated dodecylsulfate. Rojas *et al.*^23^ confirmed that dodecylsulfate has a high affinity for the LDH surface in a previous study, which mainly focused on the effect on the surface charge characteristic of LDH after dodecylsulfate was intercalated.

**Table 2:** SEM-EDX parameters of SDS-LDH composites

		SDS-LDH	Inorganic LDH
Weight (%)	Mg	10.40	21.67
	Al	3.92	7.9
	C	29.73	2.01
	S	6.51	—
	N	0.051	1.37
Atomic ratio	S/AI	1.40	—
	C/AI	17.06	0.57
	Mg/AI	2.98	3.09
	N/AI	0.025	0.33
Proposed formulae		[Mg_3_Al(OH)_7.975_(NO3)_0.025_(DDS)·4H_2_O](NaDDS)_0.4_	Mg_2.5_Al(OH)_7_(CO_3_)_0.5_·2.8H_2_O

**Fig. 2:**
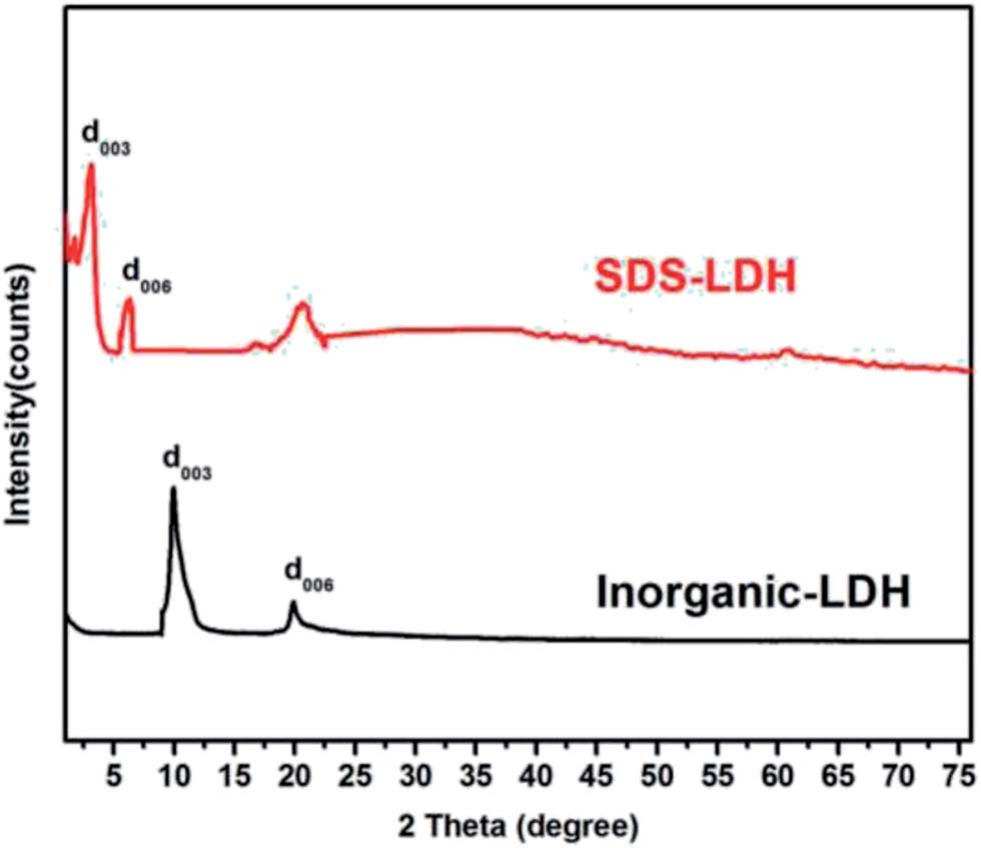
XRD spectra of SDS-LDH and inorganic LDH composites.

**Scheme 2:**
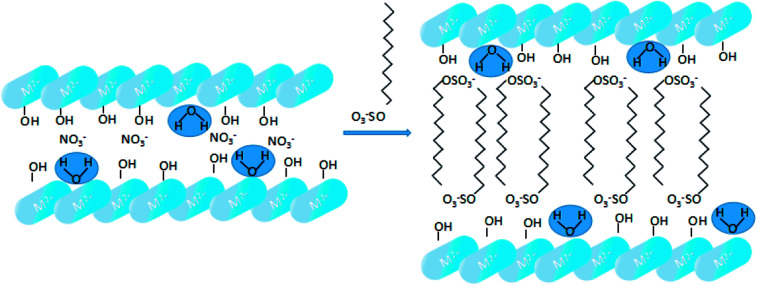
Structures of inorganic LDH composites (left) and SDS-LDH (right).

The XRD patterns of the SDS-LDH composites and inorganic LDH composites samples are displayed in Fig. 2, and the XRD parameters are listed in Table 3. Three diffraction peaks (*d*_003_, *d*_006_, and *d*_009_) typical for SDS-LDH composites appeared in the low diffraction region, and they moved forward obviously compared with the same peaks typical for inorganic LDH. The basal spacing (*d*) of the layered materials could be calculated from the basal reflection by using eqn (1). The observed basal spacings, *d*_003_, for SDS-LDH composites and inorganic LDH composites were 3.18 and 9.18 Å, and their corresponding layer spacings were 2.78 and 0.89 nm, indicating that the SDS-LDH composites inter-layer spacing was greater than that of inorganic LDH composites.^24^ The intercalated SDS-LDH composites changed the positions of the (003), (006), (012), and (110) diffraction reflections presumably because in SDS-LDH composites, the chemical composition and therefore the morphology of the LDH were changed.^25–27^ Given that the length of dodecylsulfate was 2.08 nm, it intercalated successfully into the interlayer and arranged the single layer in layers. Scheme 2 shown the structures of inorganic LDH composites (left) and SDS-LDH composites (right).

**Table 3:** XRD parameters of SDS-LDH composites

Samples	2*θ*_003_ (°)	*d*_003_ (nm)	2*θ*_006_ (°)	*d*_006_ (nm)	*d*_layer_ (nm)	*d* (nm)	*d*_SDS_ (nm)
SDS-DLH	3.18	2.78	6.29	1.40	0.49	2.29	2.08
Inorganic LDH	9.98	0.89	19.89	0.45	0.49	0.40	2.08

**Fig. 3:**
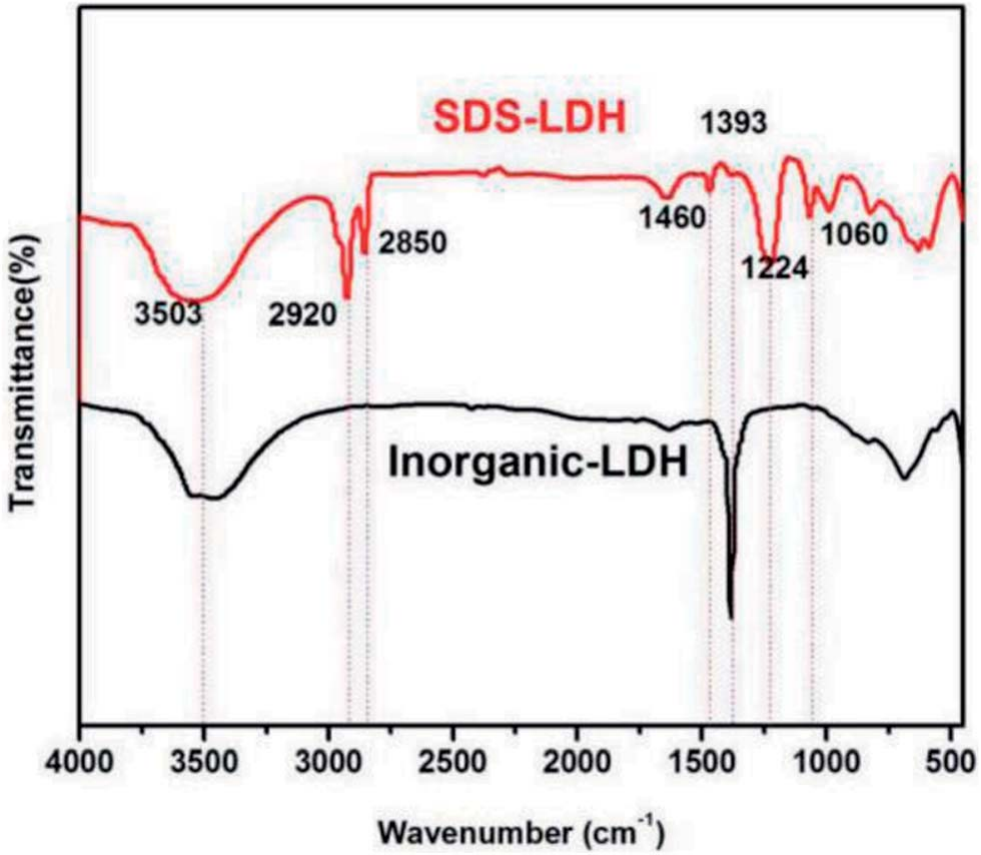
FT-IR spectra of SDS-LDH and inorganic LDH composites.

FT-IR analysis was performed to verify whether dodecylsulfate successfully intercalated into the interlayer. As shown in Fig. 3, the infrared spectra of SDS-LDH composites and inorganic LDH composites showed the characteristic absorption bands according to the hydrotalcite structure and composition. On the one hand, both of them showed a broad band centered at 3503 cm^−1^ due to the stretching mode of OH, the stretching vibration bands of the hydration water molecules, and the OH^−^ groups of the brucite-like layers. The peaks at 1393 cm^−1^ could be attributed to the vibration of NO_3_^−^. The peak intensity of SDS-LDH at this point sharply decreased compared with that of inorganic LDH. On the other hand, five peaks that belonged only to composites appeared in the spectra. The presence of dodecylsulfate in SDS-LDH composites was confirmed by the double in the 2850–2920 cm^−1^ region due to the CH stretching vibration bands (2920 and 2850 cm^−1^) and the C–H bending vibration band at 1460 cm^−1^. Bands due to the antisymmetric (1224 cm^−1^) and symmetric (1060 cm^−1^) sulfate S=O stretching vibrations were also observed.^12,28^ The correlated data are included in Table 4. The results indicated that dodecylsulfate replaced CO_3_^2−^ and NO_3_^−^ into the interlayer.

**Table 4:** FT-IR parameters of SDS-LDH composites

Wavenumber (cm^−1^)	Functional group
1224, 1060	S=O
1393	NO_3_^−^
1460	C–H
2850, 2920	–CH_3_ & –CH_2_
3503	HO⋯OH_2_ & H_2_O⋯OH_2_

### 3.2 Adsorption performance of E2 under different conditions

#### 3.2.1 Effect of pH on the adsorption of E2

**Fig. 4:**
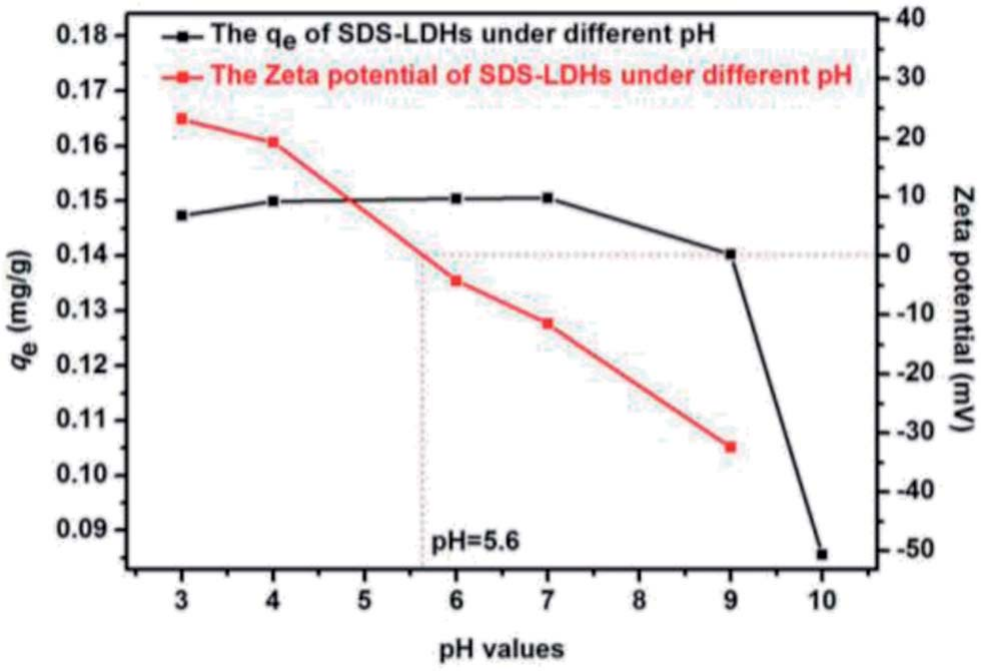
Effect of solution pH for the adsorption of 17β-estradiol onto SDS-LDH composites and zeta potential of SDS-LDH composites (dosages = 2 g L^−1^, solutions = 0.319 mg L^−1^, *V* = 200 mL, time = 45 min, temperature = 298 K).

The pH can influence the surface charge of the adsorbent material and the dissociation properties of organic contaminants.^29^ As shown in Fig. 4, the *q*_e_ of SDS-LDH remained relatively constant in the pH range of 3.0–9.0 and then rapidly decreased when the initial pH values exceeded 9.0. The zeta potential of SDS-LDH was zero when the pH = 5.6, indicating that the process of adsorption of E2 might be pH-independent and the SDS-LDH maintained a relatively high performance in both acidic and weakly basic solution. These inconsistent phenomenon between isoelectric point and *q*_e_ can be explained by hydrophobic interaction, hydrogen bond and dissociation properties of E2. When the initial pH of aqueous solution was less than 9.0, the dissociation of E2 (p*K*_a_ = 10.71) was partially restrained, causing E2 to exist mostly in molecular form,^30^ therefore, even though the electrical charge of the SDS-LDH gradually changed from positive to negative^31^ (when pH exceeded 5.6), the electrostatic repulsion would not be the major force. Under this condition, the interaction between hydroxyl (from E2) and sulfonic group (from intercalated dodecylsulfate anions), or hydroxyl (boned to layer), can formed hydrogen bond and became one of the major force during the adsorption process. Another important force that existed during the adsorption process was hydrophobic interaction, the hydrophobic phase in the interlayer region was expected to have the ability to solubilize hydrophobic organic E2 compounds (the hydrophobic properties of SDS-LDH were going to be explained in Chapter 3.4.4.1). When the initial pH exceeded 9.0, the increased hydroxyl ion in aqueous promoted the dissociation of E2, and the main form was E2 anions. Thus the electrostatic repulsion between SDS-LDH and E2 anions became the major force and *q*_e_ sharply decreased. Therefore, pH 7 was considered the optimal pH condition and used in subsequent experiments.

#### 3.2.2 Effect of adsorbent dosage on the adsorption process of E2

**Fig. 5:**
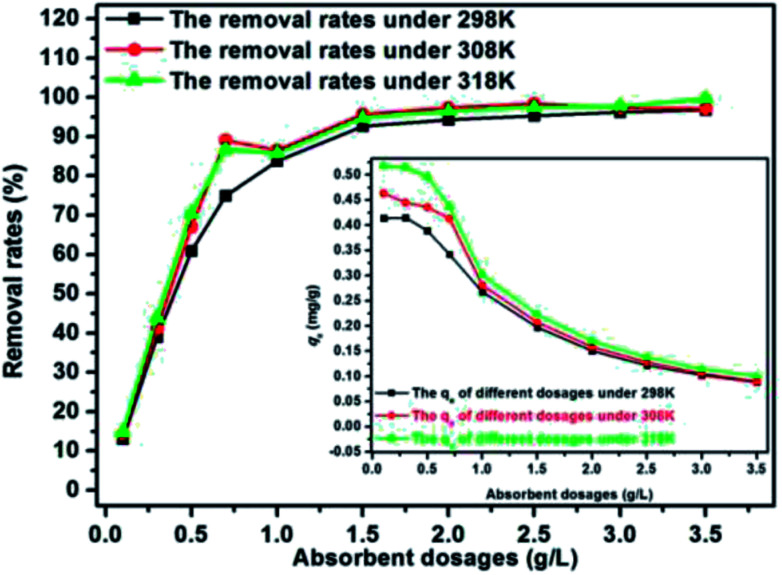
Effect of adsorbent dosage for the adsorption of 17β-estradiol on removal rate and equilibrium adsorption amount *q*_e_ (inset) (solutions = 0.320 mg L^−1^, *V* = 200 mL, pH = 7, time = 45 min, temperature = 298 K).

The experiments about the effect of adsorbent dosage can determine the appropriate dosage of adsorbent, and the results can be used to explore the adsorption isotherms. The results under different temperature were presented in Fig. 5. It shown that the variation tendency in all three kind of experimental temperature (298 K, 308 K, 318 K) are similar. One the one hand, the removal rate of E2 increased monotonously as the adsorbent dosage increased before reaching a maximum removal (dosage less than 2 g L^−1^), and then the removal rate changed slightly, indicating that the adsorption process reached equilibrium. This could be explained by the increased adsorption site with more SDS-LDH used. On the other hand, the *q*_e_ of SDS-LDH was gradually decreased with more SDS-LDH used. Therefore, 2 g L^−1^ was considered the optimal dosage and used in subsequent experiments.

#### 3.2.3 Effect of ions on the adsorption of E2

**Fig. 6:**
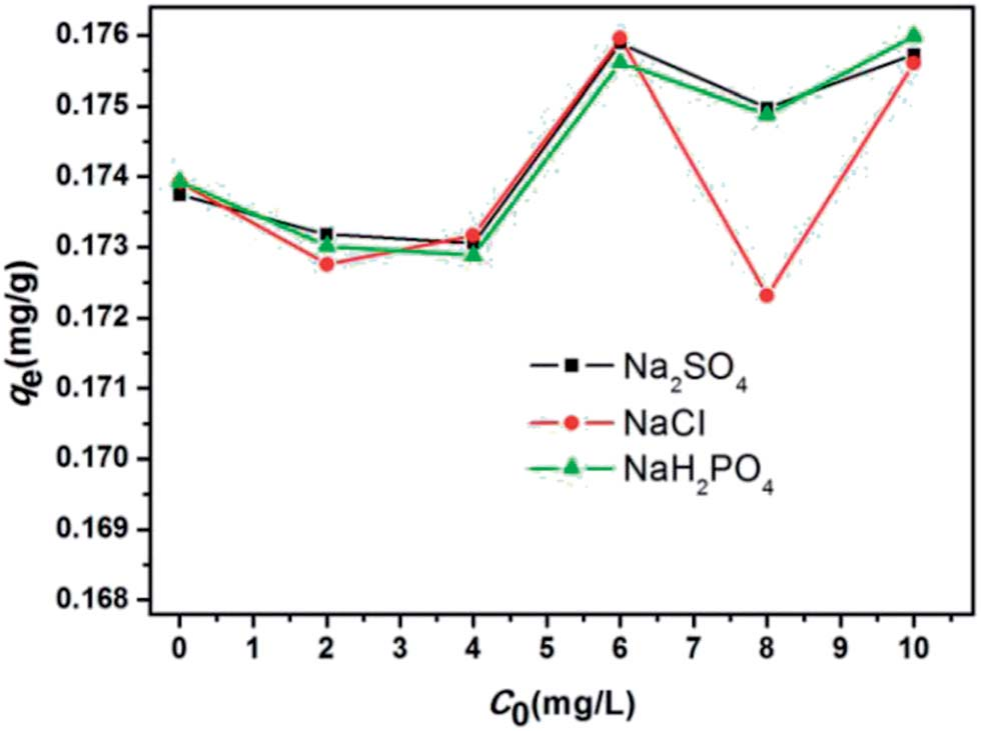
Effect of ion strength for the adsorption of 17β-estradiol onto SDS-LDH composites (dosages = 2 g L^−1^, solutions = 0.379 mg L^−1^, *V* = 200 mL, pH = 7, reaction time = 45 min, *T* = 298 K).

Given that many ions existed in wastewater and that these ions participated in the adsorption process between absorbents and pollutants and consequently affected the functional groups on the adsorbent surface, ion strength was considered a significant factor in aqueous solution. As shown in Fig. 6, no obvious change occurred when the concentrations of Na_2_SO_4_, NaCl, and NaH_2_PO_4_ were gradually increased. Thus, the ions did not compete with the E2 molecules adsorbed onto the absorbents. This finding, together with the previous experiment on the effect of pH on the adsorption process, revealed that the adsorption of E2 onto adsorbents exhibited pH dependence and ion independence.

#### 3.2.4 Effect of reaction time on the adsorption of E2

**Fig. 7:**
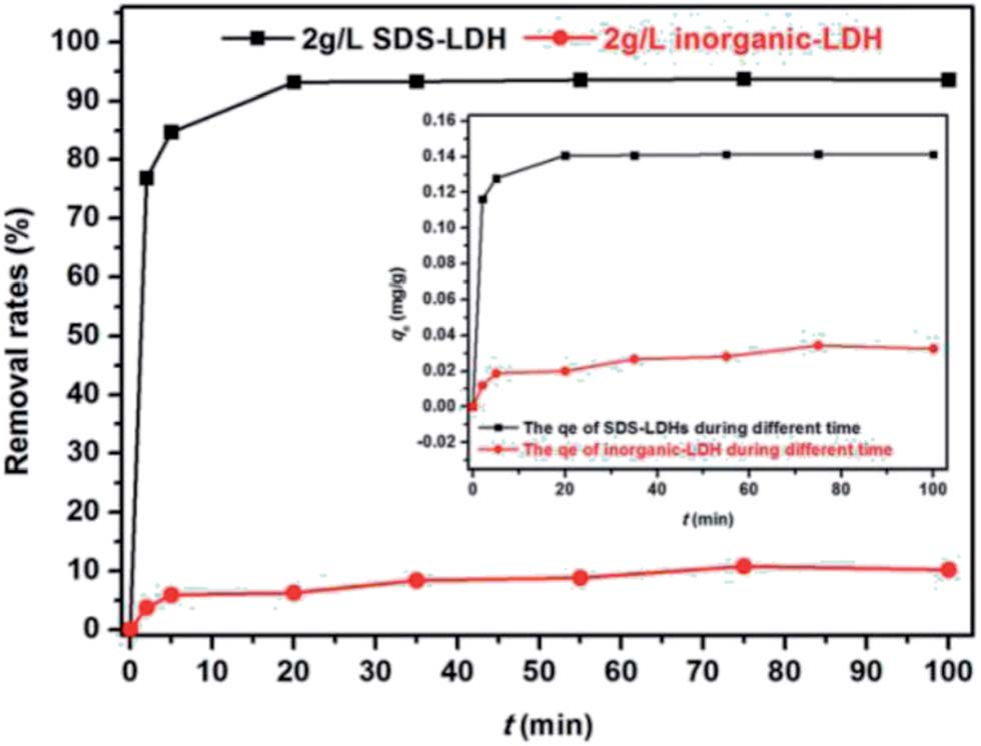
Effect of contact time for the adsorption of 17β-estradiol on removal rates and the adsorption amount *q*_*t*_ (inset) (dosages = 2 g L^−1^, solutions = 0.302 mg L^−1^, *V* = 500 mL, pH = 7, *T* = 298 K).

The effect of contact time is presented in Fig. 7. The removal rate of E2 by SDS-LDH composites reached 94%, whereas that by inorganic LDH composites was only 10%. The adsorption process of SDS-LDH composites was rapid in the initial 3 min and then gradually slowed down compared with that of inorganic LDH. The adsorption equilibrium was reached within 20 min. This phenomenon can be explained by the adsorption and desorption equilibrium. Numerous sorption sites initially appeared on the adsorbent surface. Thus, the adsorption rate was fast. Given that the adsorption sites on the surface were increasingly occupied by E2 during the adsorption process, the adsorption rate gradually decreased, and the desorption rate gradually increased. Then, the E2 molecules diffused into the interlayer of the adsorbents and were adsorbed through hydrogen bonding interactions with the sulfonic group belonging to the intercalated dodecylsulfate anions or hydroxyl of bound water and metal hydroxide. The E2 molecules were also adsorbed through hydrophobic interactions with the hydrophobic alkyl groups of intercalated dodecylsulfate, this process required a relatively long time. Finally, SDS-LDH composites was saturated when the desorption rate was equal to the adsorption rate. Moreover, the apparent adsorption rate of SDS-LDH composites was faster than that of inorganic LDH composites. These observations implied that intercalating dodecylsulfate into the interlayer could enhance the hydrophobicity of SDS-LDH composites and intensify the hydrophobic interaction, thereby accelerating the adsorption process compared with that of inorganic LDH composites.

#### 3.2.5 Effect of temperature on the adsorption process of E2

**Fig. 8:**
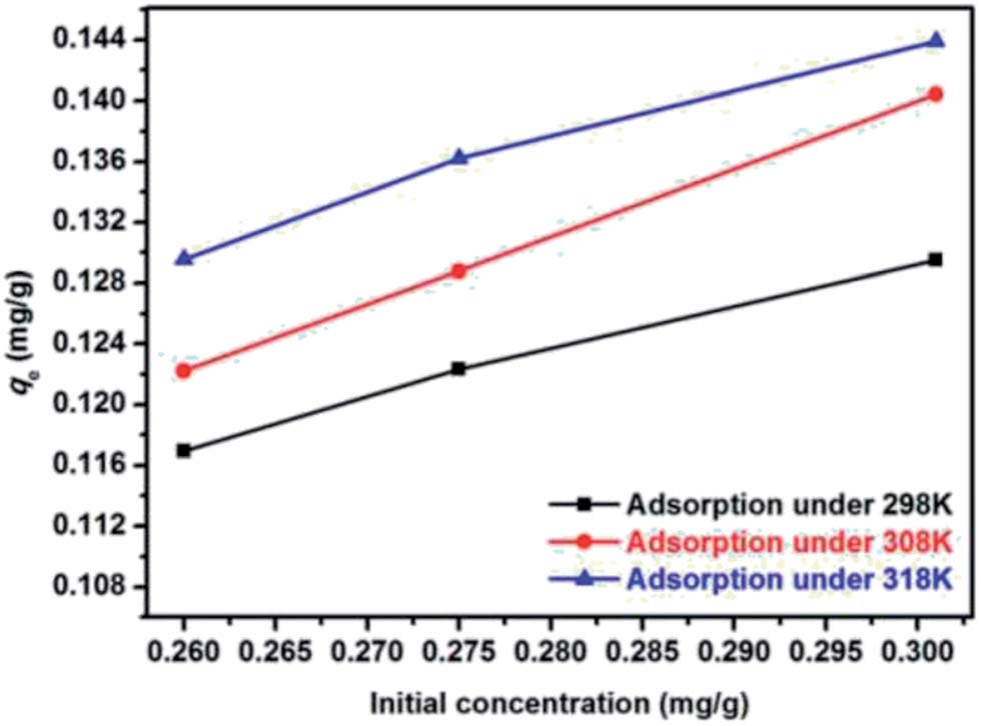
Effect of temperature on the adsorption process (dosages = 2 g L^−1^, solutions = 0.260 mg L^−1^; 0.275 mg L^−1^; 0.301 mg L^−1^, *V* = 200 mL, pH = 7, reaction time = 45 min).

The effect of temperature was presented in Fig. 8 and qe was promoted as the temperature increased. when the temperature was increased from 298 K to 308 K and 318 K, the *q*_e_ of the SDS-LDH composites increased from 0.130 mg g^−1^ to 0.140 and 0.144 mg g^−1^ with the initial E2 concentration was 0.301 mg L^−1^. This phenomenon suggested that the adsorption processes were endothermic and higher temperatures were favorable to the adsorption process as higher temperatures increased the diffusion rate of the adsorbent (SDS-LDH) and adsorbate (E2) in solution. On the other hand, E2 obtains more energy to overcome the repulsive force with the SDS-LDHs. Therefore, as the temperature increases, the rate of adsorption reaction increases.

### 3.3 Adsorption performance of TP, PO_4_^3−^ compounds

#### 3.3.1 Comparison of the adsorption performances of PO_4_^3−^ between SDS-LDH composites and inorganic LDH composites

**Fig. 9:**
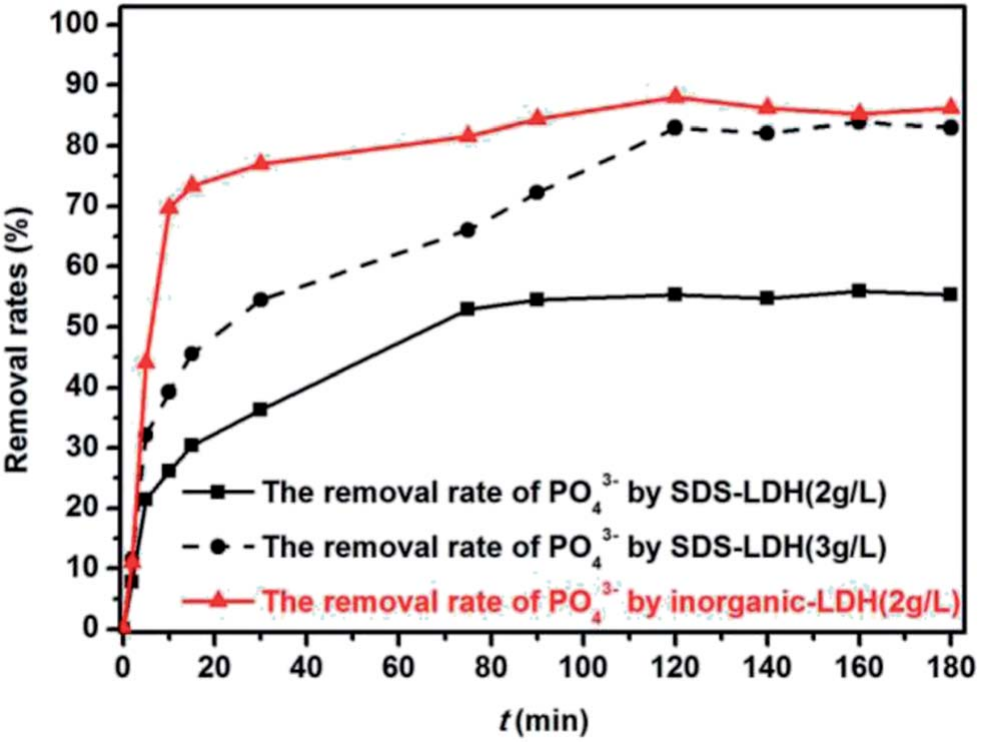
Effect of contact time for the adsorption of PO_4_^3−^ on removal rate (dosages = 2 g L^−1^ for SDS-LDH and inorganic-LDH; 3 g L^−1^ for SDS-LDH, *C*_TP_ = 2.952 mg L^−1^, *C*_PO_4_^3−^_ = 2.379 mg L^−1^, V = 200 mL, pH = 7, reaction time = 180 minutes, *T* = 298 K).

As shown in Fig. 9, the adsorption process of PO_4_^3−^ onto the adsorbents was rapid, especially in the initial 10 min. After which, the process gradually slowed down. The adsorption equilibrium was reached within 120 min. This phenomenon was similar to the preceding experiments and has been explained. Moreover, the removal rates of PO_4_^3−^ by the SDS-LDH composites reached 85% when the equilibrium time and dosage were 80 min and 3 g L^−1^, respectively. Almost the same removal rate was attained by inorganic LDH at an equilibrium time and dosage of 30 min and 2 g L^−1^, respectively. These results indicated that the removal capacity of the SDS-LDH composites for the adsorbed PO_4_^3−^ was slightly reduced after the dodecylsulfate anions intercalated into the interlayer. This phenomenon can be presented in two aspects, namely, contact time and dosage. The data suggested that the hydrophobicity of the adsorbent composites was markedly enhanced after dodecylsulfate anions were intercalated into the interlayer, thereby decreasing the adsorptive capacity to hydrophilic substances, such as PO_4_^3−^.

#### 3.3.2 Removal rates of TP, PO_4_^3−^ by SDS-LDH

**Fig. 10:**
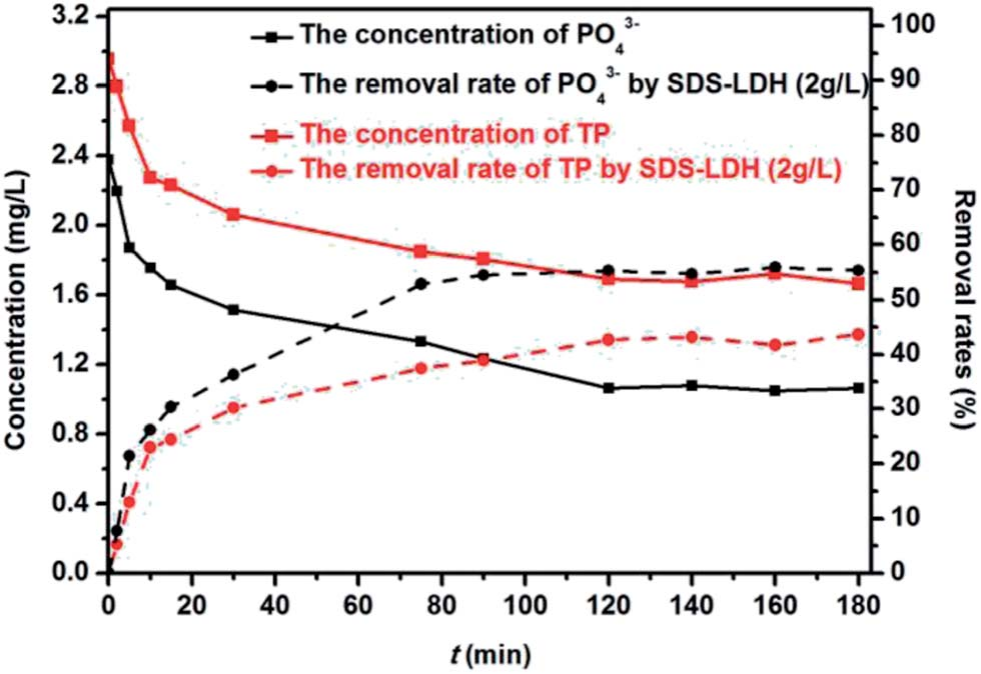
Removal rates of TP, PO_4_^3−^ by SDS-LDH composites (*C*_SDS-LDH_ = 2 g L^−1^, *C*_TP_ = 2.952 mg L^−1^, *C*_PO_4_^3−^_ = 2.379 mg L^−1^, *V* = 200 mL, pH = 7, reaction time = 180 min, *T* = 298 K).

Fig. 10 shows the changes in the contents of TP, PO_4_^3−^, and organophosphorus. The removal rates of TP and PO_4_^3−^ were 65.45% and 83.01%, respectively, whereas the concentration of organophosphorus remained nearly constant. This phenomenon indicated that the SDS-LDH composites mainly removed PO_4_^3−^ to reduce the contents of TP.

### 3.4 Adsorption theory discussion

#### 3.4.1 Adsorption kinetics

Adsorption kinetics reflects the adsorbent efficiency and application potential of modified materials. Adsorption kinetics is generally used to describe the dynamic behavior and explore the adsorption mechanism. To investigate the uptake kinetics of E2 onto SDS-LDH and to examine the mechanism and rate-controlling steps in the overall adsorption process, pseudo-first order and pseudo-second order kinetics models was adopted to fit the experimental data.

**Fig. 11:**
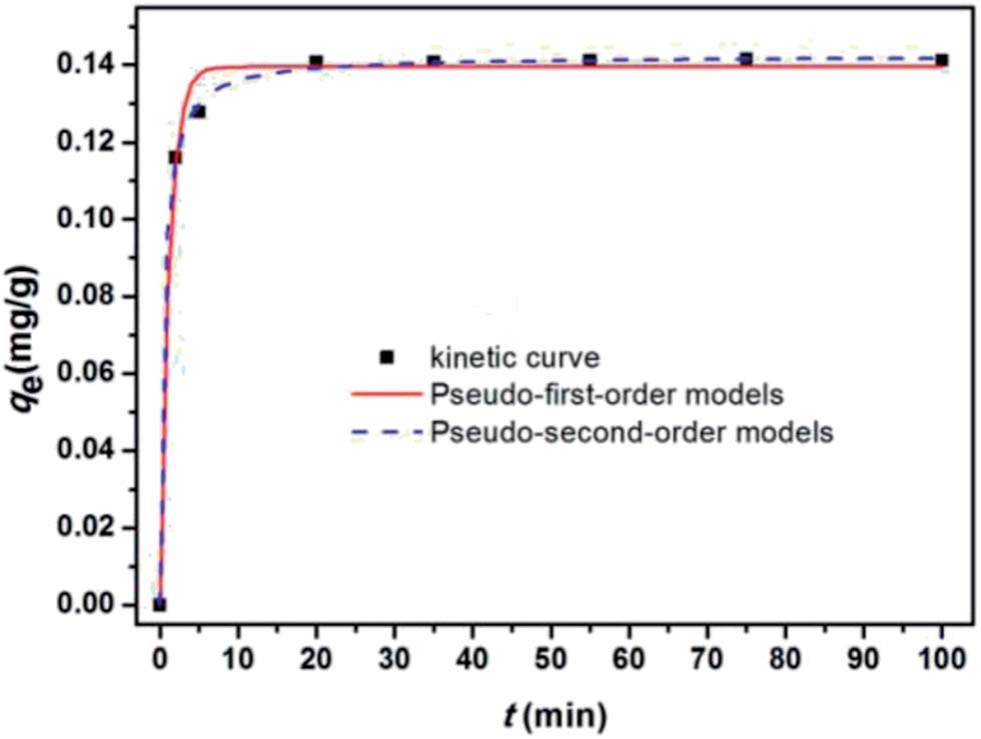
Nonlinear fitting of the pseudo-first-order and pseudo-second-order models for the adsorption of E2 onto SDS-LDH composites.

Fig. 11 show the kinetic curves of E2, corresponding results of the fitting models. Parameters *q*_e_, *k*, and the correlation coefficient (*R*^2^) are listed in Table 5. As shown in Table 5, the pseudo-second-order model exhibited a higher correlation coefficient (*R*^2^) for E2, and the equilibrium adsorption amounts (*q*_e2(cal)_) calculated by the pseudo-second-order model were approximately identical to the experimental data (*q*_e(exp)_). These results implied that the adsorptive reaction of E2 onto SDS-LDH composites could be appropriately described by the pseudo-second-order model and that the adsorption rate was proportional to the square of the number of free sites.

**Table 5:** Parameters fitted by the pseudo-first-order and pseudo-second-order models

Material	Pollutant	*q*_e(exp)_ (mg g^−1^)	Pseudo-first-order kinetic model			Pseudo-second-order kinetic model		
			*k*_1_ (s^−1^)	*q*_e1(cal)_ (mg g^−1^)	*R* ^2^	*k*_2_ (g mg^−1^ s^−1^)	*q*_e2(cal)_ (mg g^−1^)	*R* ^2^
SDS-LDH	E2	0.141	0.85	0.139	0.99	14.85	0.142	1
	TP	0.644	0.06	0.605	0.97	0.12	0.673	0.99

#### 3.4.2 Adsorption isotherm

Adsorption isotherms are typically used to describe the adsorption characteristics, including adsorption capacity, adsorption strength, and adsorption state. Adsorption capacity is an important factor for evaluating SDS-LDH composites. Two common models are the Langmuir model and Freundlich model. The Langmuir model is based on the ideal assumption: it supposes that the adsorbent surface is completely homogeneous with identical adsorption sites and that the adsorption is a monolayer coverage according to the assumption. The Freundlich model is an empirical equation that assumes a heterogeneous adsorptive energy and is not restricted to the formation of a monolayer on the adsorbent surface.

**Fig. 12:**
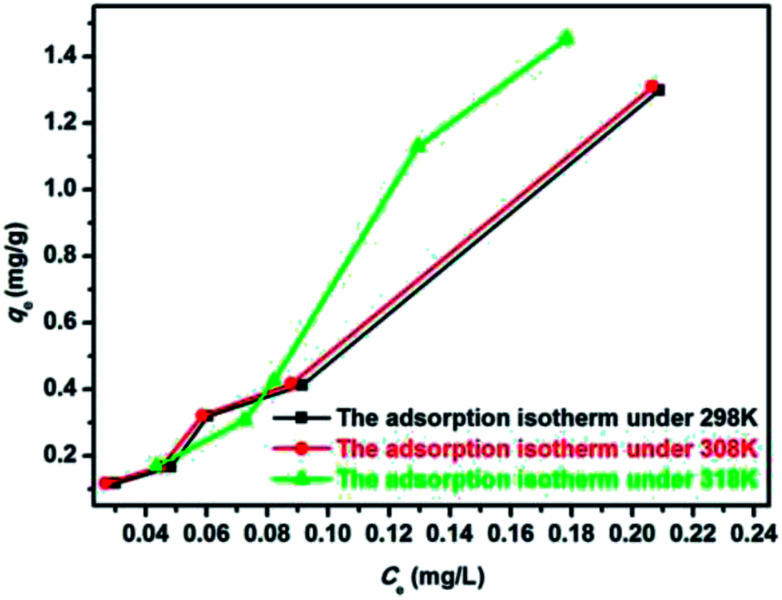
Adsorption isotherm for the adsorption of E2 onto SDS-LDH composites.

**Fig. 13:**
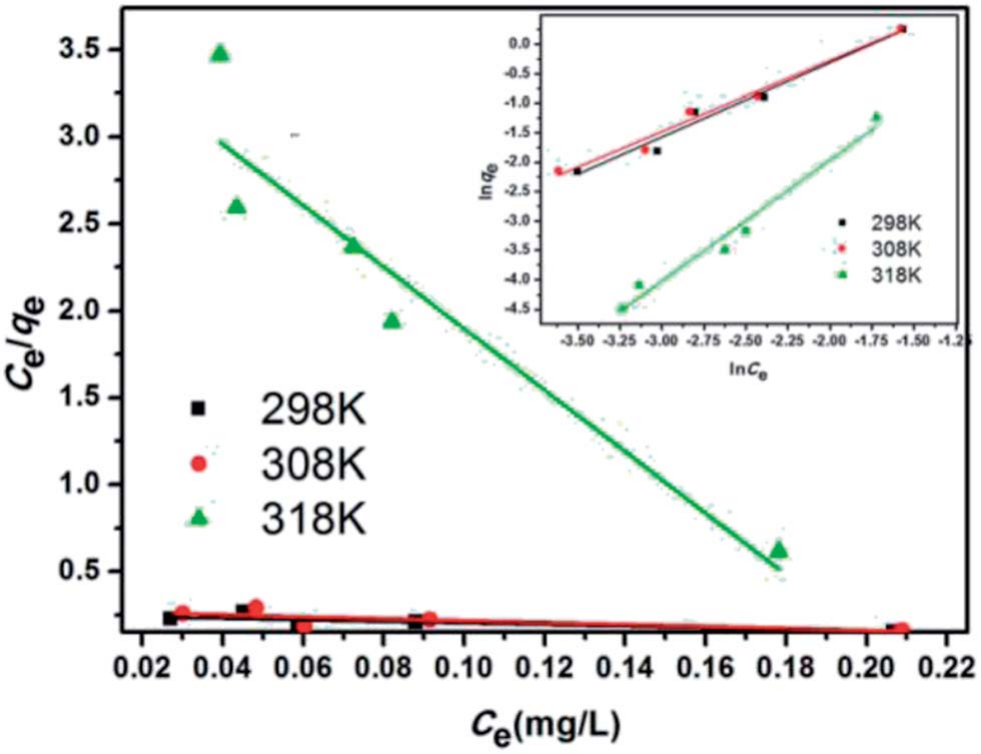
Linear fitting of the Langmuir and Freundlich (inset) models for the adsorption of E2 onto SDS-LDH composites.

Fig. 12 and 13 show the adsorption isotherm curves of E2 under different temperatures and the corresponding linear fitting of the Langmuir and Freundlich (inset) models. Parameters *Q*_m_, *K*_L_, *n*, *K*_f_, and *R*^2^ are listed in Table 6.

**Table 6:** Parameters fitted by the Langmuir and Freundlich models

Materials	Pollutant	*T* (K)	Langmuir model			Freundlich model		
			*K*_L_ (×10^−4^) (L mg^−1^)	*Q*_m_ (mg g^−1^)	*R* ^2^	*n*	*K*_f_ (mg^1−1/*n*^ L^1/*n*^ g^−1^)	*R* ^2^
SDS-LDH	17β-Estradiol	298 K	0.85	67 698.86	0.94	0.77	9.80	0.99
		308 K	1.37	43 525.36	0.96	0.80	9.40	0.99
		318 K	2.69	5109.62	0.70	0.52	8.32	0.91

As shown in Table 6, the Freundlich model exhibited a higher *R*^2^ for the adsorption process, indicating that the Freundlich equation provided a better fit for the experimental data than the Langmuir equation. The Langmuir isotherm is typically applicable to a homogeneous uptake surface where all of the uptake sites have an equal adsorbate affinity, whereas the Freundlich isotherm model assumes the heterogeneity of the uptake surfaces expressed by the Freundlich equation. Adsorption type is not restricted to the formation of a monolayer, and the adsorption sites are energetically unequal. Moreover, the higher *K*_f_ values indicated that E2 had a high affinity for SDS-LDH composites. This phenomenon implied that the surface nature of the SDS-LDH composites transformed from hydrophilic to hydrophobic after dodecylsulfate anions were intercalated into the interlayer and replaced the interior NO_3_^−^ and CO_3_^2−^, thereby increasing its affinity for hydrophobic compounds, such as E2, through adsolubilization mechanisms.

Basing on the initial slope of the isothermal adsorption curve, B. Wang *et al.*^16^ divided the liquid single component adsorption isotherm into four types, namely, types S, L, H, and C, and each type was divided into five groups. The adsorption isotherm curves obtained under different temperatures in this experiment were close to type C, which is a common adsorption isotherm curve for very hydrophobic organic compounds. This finding demonstrated that the competitive adsorption between the solvent and the adsorbate onto the binding sites of the adsorbents was minimal and that the adsorbate had a certain proportion of distribution in the solvent and the adsorbent, reflecting a constant distribution mechanism.

#### 3.4.3 Adsorption thermodynamics

Adsorption thermodynamics is generally used to determine whether an adsorptive reaction can occur spontaneously in the real process by evaluating the Gibbs free energy change, which is considered a basic standard for assessing spontaneity. Adsorption thermodynamics can also be applied to assess the effect of temperature by evaluating the heat change of the adsorption reactions.

The thermodynamic parameters of Δ*H*, Δ*S*, Δ*G*, and *R*^2^ are listed in Table 7. As shown, the adsorption processes were endothermic, and higher temperatures were conducive to the adsorptive reaction, as Δ*H* of the SDS-LDH composites was 47.39 KJ mol^−1^. The Δ*S* of the SDS-LDH composites was 173.80 J mol^−1^ K^−1^. Δ*G* at 298, 308, and 318 K were calculated as −4.33, −6.48, and −7.81 kJ mol^−1^, respectively. These data confirmed that the adsorbent particles had a good affinity toward E2 and that the adsorption process occurred spontaneously under natural conditions.

**Table 7:** Thermodynamic parameters for the adsorption processes

Material	*C*_0_ (mg L^−1^)	*T* (K)	Δ*S* (J mol^−1^ K^−1^)	Δ*H* (kJ mol^−1^)	Δ*G* (kJ mol^−1^)
SDS-LDH	0.30143	298			−4.33
		308	173.80	47.39	−6.48
		318			−7.81

#### 3.4.4 Adsorption mechanism

##### 3.4.4.1 Hydrophobicity of SDS-LDH

**Scheme 3:**
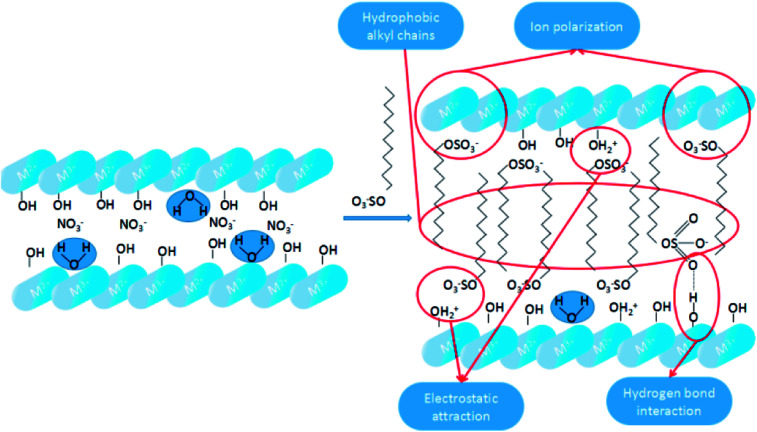
Illustration for the hydrophobic of SDS-LDH composites.

As shown in Scheme 3, the dodecylsulfate anions were assembled into the LDH interlayers through electrostatic attraction and hydrogen bonding interaction. According to the XRD characterization of the SDS-LDH composites, the dodecylsulfate anions existed in a single-layer vertical pattern among the lamella. The hydrophobic properties of SDS-LDH composites can be explained from two aspects. One is ion polarization, the ionic radius of –OSO_3_– is large and –OSO_3_– has poor binding force for the outer electrons, which make the outer electrons prone to deformity. On the contrast, the ionic radii of Mg^2+^ and Al^3+^ were relatively small and occupied with high positive charges. Consequently, these metallic cation could induced relative displacement between the outer electrons of –OSO_3_– and nucleus, and then ion polarization occurred which means the covalent component of SDS-LDH composites was increased compared with that of the inorganic LDH composites, and the solubility in water would inevitably decrease. Another reason is the function of hydrophobic dodecylsulfate anions, the alkyl chains (from dodecylsulfate anions) occupied the interlayer regions and formed a compact and ordered structure,^32^ resulting in the formation of some hydrophobic phase existing in the interlayer region.

##### 3.4.4.2 Mechanism of the adsorption process of E2

**Scheme 4:**
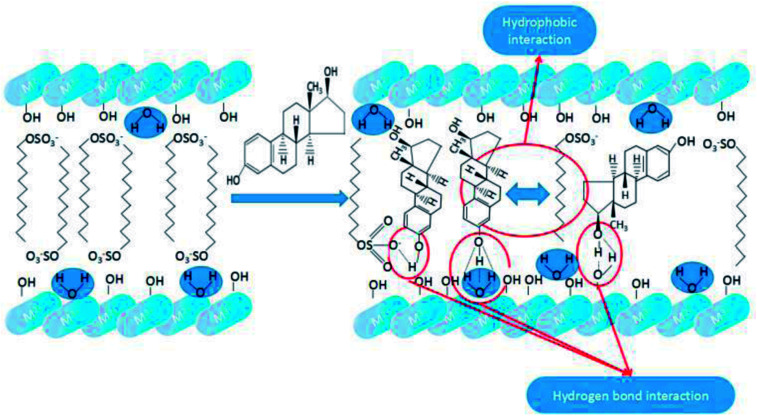
Mechanistic illustration for the adsorption of E2 onto SDS-LDH composites.

As shown in Scheme 4, both hydrogen bond interaction and hydrophobic interaction between E2 and SDS-LDH composites were the main force during the process of adsorption. When the pH values of aqueous solution was less than 9.0, the electrostatic repulsion would not be the major force affecting the adsorption process due to that E2 (p*K*_a_ = 10.71) was mainly existed in molecule state within this PH range. Under this situation, the interaction between hydroxyl (from E2) and sulfonic group (from intercalated dodecylsulfate anions) can formed hydrogen bond which was regarded as one of the major force during the adsorption process.^33^ Based on the explanation for hydrophobic of SDS-LDH composites in Chapter 3.4.4.1, another important force during the process of adsorption was adsolubilization.^34,35^ Namely, the aggressive of the dodecylsulfate anions especially some hydrophobic alkyl chains in the SDS-LDH composites interlayers would result in some hydrophobic phase and that hydrophobic phase was expected to have the ability to solubilize hydrophobic organic compounds.^32,34,35^ When the pH values was exceeded 9.0, E2 was mainly in the form of anion, under this situation, the intense electrostatic repulsion keep the ionic state-E2 away from the SDS-LDH composites and adsorption efficiency was inevitable decreased.

### 3.5 Recycle and reuse of SDS-LDH composites

The regeneration performance of adsorbents is a critical factor in evaluating the practicability of adsorbents and improving the process economics. The experimental results for solution pH, where the adsorption process of E2 onto the adsorbents was restrained at high alkali conditions, suggested that the adsorbed SDS-LDH composites were highly likely to be regenerated by alkali treatment.

**Fig. 14:**
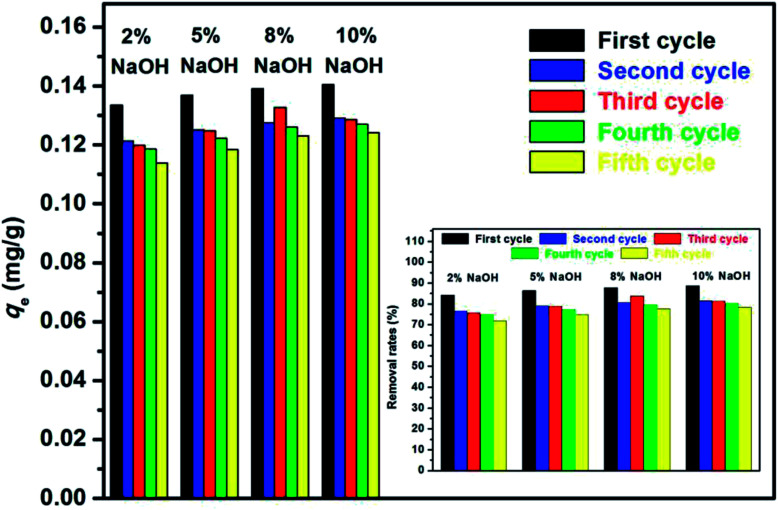
Effect of recycling time for the adsorption of E2 on the equilibrium adsorption amount *q*_e_ and the removal rate (inset).

Adsorption–desorption cycles were repeated five times. To compare the efficiency of the treatment under different concentrations of NaOH, the cyclic adsorption experiments were divided into four groups, which were occupied with 2%, 5%, 8%, 10% NaOH, respectively, and marked as groups A, B, C, and D. The adsorbed materials of each group were treated with the corresponding concentrations of NaOH alkali solution. The results are shown in Fig. 14.

Alkaline treatment achieved a good regeneration performance of the adsorbents. The *q*_e_ values of the initial adsorption of groups A, B, C, and D were 0.133, 0.137, 0.139, and 0.140 mg g^−1^, respectively, and their corresponding removal rates were 84.19%, 86.34%, 87.71%, and 88.60%. After five times of recycle, the *q*_e_ values were measured as 0.114, 0.118, 0.123, and 0.124 mg g^−1^, respectively, and their corresponding removal rates were 71.84%, 74.72%, 77.63%, and 78.28%. The results revealed that the regenerated adsorbents showed an excellent reusability and stability with only a slight decrease in *q*_e_. Moreover, group D exhibited the best regeneration performance among these groups, indicating that the efficiency of the treatment was improved as the NaOH concentration was increased within the limits.

## 4 Conclusion

SDS-LDH composites were successfully synthesized by intercalating dodecylsulfate anions into the interlayer of LDH. The formed material exhibited a spreading lamellar structure, and its layers were thin and diffused irregularly, which were conducive for a superb adsorption performance toward trace-level E2. The maximum adsorption equilibrium amount and the adsorption equilibrium time at 298 K were 0.142 mg g^−1^ and 20 min, respectively. The suitable pH and adsorbent dosage conditions for absorbing E2 were 7 and 2 g L^−1^, respectively. The presence of various ions in aqueous solution (Na^+^, SO_4_^2−^, CI^−^, and H_2_PO_4_^−^) did not adversely affect the adsorption process. The pseudo-second-order model was better fitted with the kinetic data than the pseudo-first-order model, and the Freundlich isotherm was better correlated than the Langmuir isotherm. The adsorption processes were spontaneous and endothermic, and an excellent recycle was obtained in the cyclic adsorption experiments. Moreover, the removal capacity of SDS-LDH composites for inorganic ion (PO_4_^3−^) was reduced and presented in two aspects (contact time and dosage) compared with that of the inorganic LDH composites.

Therefore, we believe that SDS-LDH is a technically feasible, easily synthesizable, highly efficient, stably functional, and cost-effective adsorbent and it could be applied in the treatment of secondary effluent of wastewater treatment plants contaminated by trace-level E2 compounds.

## Conflicts of interest

There are no conflicts to declare.

## Supplementary Material
